# Stronger connectivity and higher extraversion protect against stress-related deterioration of cognitive functions

**DOI:** 10.1038/s41598-021-96718-5

**Published:** 2021-08-31

**Authors:** Jacek Rogala, Joanna Dreszer, Urszula Malinowska, Marek Waligóra, Agnieszka Pluta, Ingrida Antonova, Andrzej Wróbel

**Affiliations:** 1 Bioimaging Research Center, World Hearing Center, Institute of Physiology and Pathology of Hearing, Warsaw, Poland; 2 The Center for Systemic Risk Analysis, Faculty of “Artes Liberales”, University of Warsaw, Warsaw, Poland; 3 Institute of Psychology, Faculty of Philosophy and Social Sciences, Nicolaus Copernicus University in Toruń, Toruń, Poland; 4 Instytut Biologii Doświadczalnej Im. Marcelego Nenckiego, Warsaw, Poland; 5 Faculty of Psychology, The University of Warsaw, Warsaw, Poland; 6 Institute of Philosophy, Faculty of Epistemology, University of Warsaw, Warsaw, Poland

**Keywords:** Cognitive neuroscience, Personality, Stress and resilience

## Abstract

Here we attempted to define the relationship between: EEG activity, personality and coping during lockdown. We were in a unique situation since the COVID-19 outbreak interrupted our independent longitudinal study. We already collected a significant amount of data before lockdown. During lockdown, a subgroup of participants willingly continued their engagement in the study. These circumstances provided us with an opportunity to examine the relationship between personality/cognition and brain rhythms in individuals who continued their engagement during lockdown compared to control data collected well before pandemic. The testing consisted of a one-time assessment of personality dimensions and two sessions of EEG recording and deductive reasoning task. Participants were divided into groups based on the time they completed the second session: before or during the COVID-19 outbreak ‘Pre-pandemic Controls’ and ‘Pandemics’, respectively. The Pandemics were characterized by a higher extraversion and stronger connectivity, compared to Pre-pandemic Controls. Furthermore, the Pandemics improved their cognitive performance under long-term stress as compared to the Pre-Pandemic Controls matched for personality traits to the Pandemics. The Pandemics were also characterized by increased EEG connectivity during lockdown. We posit that stronger EEG connectivity and higher extraversion could act as a defense mechanism against stress-related deterioration of cognitive functions.

## Introduction

A personality trait is a stable psychological characteristic that influences an individual’s thoughts, feelings, and behavior^1^. Traits such as neuroticism and extraversion appear to be key factors that predict adherence to health measures. Individuals who score high on neuroticism are often worried about their health^2^ and are more likely to maintain healthy habits^3,4^. In contrast, extraverts seek social engagements and their neural networks are activated to a greater extent by external stimuli^5,6^.

Investigations tempting to define the relationship between brain activity and personality focused on resting-state and connectivity as the potential explanatory factors. Several studies have suggested an interrelation between personality traits and patterns of whole brain resting state functional connectivity^7,8^, although these results have been challenged (for review see^9^). By contrast, it is generally accepted that individual differences in cognitive performance are mediated by differences in dynamical neural systems and brain-wide interactions. Dynamic responses of resting-state networks affect memory performance^10,11^ and visual attention^12–14^. Importantly, both memory and attention influence adherence to COVID-19 pandemic regulations^15^ and vulnerability to stress^16,17^, a finding which could be related back to individual variations in brain connectivity. Indeed, several recent studies have found stress coping strategies are related to resting state functional connectivit^y^^18,19^. Improved understanding about which inter-individual factors shape behavioral responses to perceived threats is crucial for predicting and developing relevant actions mitigating unwanted and risky behavior.

Here we attempted to define the relationship between EEG activity and coping during lockdown. We were in a unique situation since the COVID-19 outbreak interrupted our independent longitudinal EEG neurofeedback study. We had already collected a significant amount of control data well before lockdown. During lockdown, a subgroup of participants willingly continued their engagement in the study. This unique set of circumstances provided us with an opportunity to examine the relationship between personality/cognition and associated brain rhythms in individuals who continued their engagement during lockdown compared to control data collected well before pandemic.

## Results

We compared personality dimensions, cognitive function, and EEG between subjects who continued their training during the COVID-19 pandemic and those who did not. The study design included: a “Pre-pandemic Controls” who completed two test sessions (EEG recording and cognitive tasks) before the COVID-19 outbreak. The “Pandemics” had their first session before the COVID-19 outbreak and the second during lock-down. Both groups completed psychological assessments before the pandemic, and a questionnaire about fear of COVID-19 during lockdown.

### Characteristics and comparison of the Pre-pandemic Control vs Pandemic group

#### Fear of COVID-19 and Socioeconomic Status (SES)

The results of the fear of COVID-19 questionnaire are presented in Suppl. Inf. Table S1. A two-tailed Wilcoxon test did not show a difference between the fear medians (*p* = 0.76).

In our study, we also examined SES and found the dominant group of subjects were people with higher SES in both Pre-pandemic and Pandemic groups. Group statistics of SES are shown in Suppl. Inf. Table S2.

#### Personality dimensions

**Figure 1:**
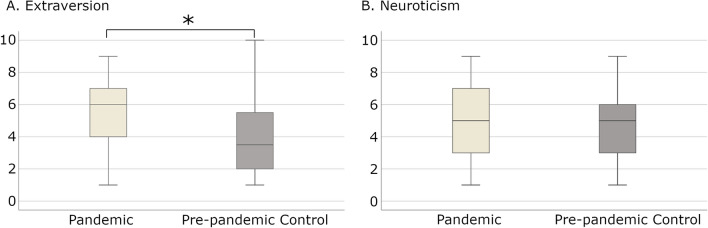
Comparison of level of extraversion (**A**) and neuroticism (**B**) on the sten score scale between Pandemic and Pre-pandemic Control groups in Session-1. The asterisk denotes a significant difference between groups (*p* < 0.05 Mann–Whitney test). Created using R 4.0.4 (https://www.r-project.org).

During Session-1 (before pandemic), we found a higher extraversion score (*p* < 0.05, Mann–Whitney U test) in the Pandemic group compared to the Pre-pandemic Control (Fig. 1A) and no differences for neuroticism scores (Fig. 1B).

#### Global connectivity index

**Figure 2:**
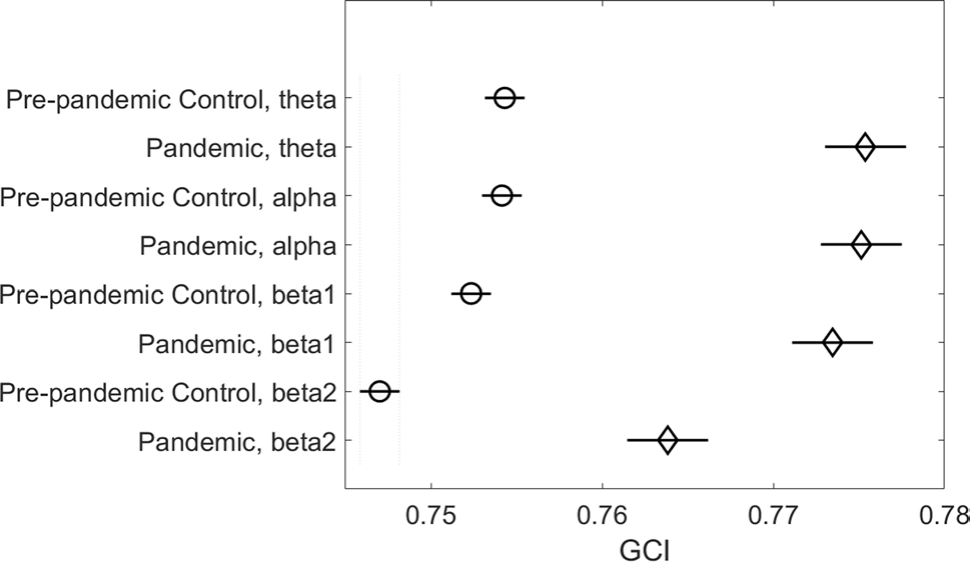
Group means of Global Connectivity Index in four EEG bands during Session-1 (before COVID-19 pandemic). Between-group differences in all bands were significant (*p* < 0.01, ANOVA followed by Tukey post-hoc test). GCI was calculated as the averaged phase locking value (the measure of connectivity was based on the phase of the EEG signal) in the four canonical EEG bands: theta (4-7 Hz), alpha (8–12 Hz), beta-1 (14–20 Hz) and beta-2 (21–30 Hz). Created using MATLAB 2020a (The MathWorks, Inc, www.mathworks.com).

Higher extraversion scores of the Pandemics, led us to speculate that biopsychological differences proposed by Eysenck^6^ might be detectable in EEG. We analyzed differences in connectivity using the Global Connectivity Index (GCI; see Fig. 2 legend), as similar measures appeared to be a better predictor of cognitive performance than detailed connectivity metrics^20^.

Analyses of the GCI for Session-1 using a two factor ANOVA for group (Pre-pandemic Control, n = 44 and Pandemic, n = 18) and EEG band (theta, alpha, beta-1, beta-2) revealed a significant effect of group (F = 497.59, *p* < 0.01) and EEG band (F = 71.74, *p* < 0.01) but no significance for interaction (F = 1.79, *p* = 0.15). Subsequent Tukey–Kramer post-hoc testing revealed significantly higher (*p* < 0.01) GCI values in the Pandemic group compared to controls for all EEG bands (Fig. 2).

**Figure 3:**
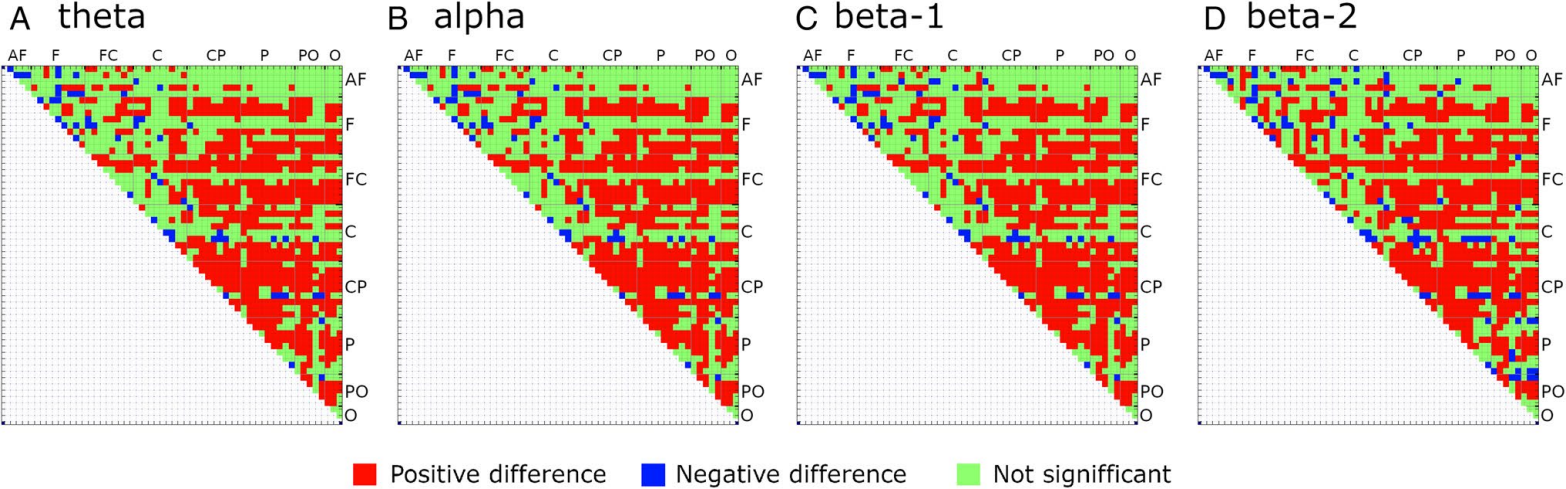
PLV differences between Pandemic and Pre-pandemic Control groups in Session-1. Green squares denote a lack of significance. Differences significant at *p* < 0.01, Bonferroni corrected. Abbreviation for regions with appropriate electrodes: AF – anterofrontal; F – frontal; C – central; CP – centroparietal; P – parietal; PO – posterooccipital; O – occipital. Created using MATLAB 2020a (The MathWorks, Inc, www.mathworks.com).

Given the differences between the groups in GCI, we examined connectivity using mean PLVs for all pairs of electrodes (matrix 57X57) in all EEG bands. The resulting connectivity matrix can be used to assess whether GCI differences are local—spread over specific head areas or global. Additionally, the strength of EEG connectivity between frontal and parietal brain areas has recently been shown to be related to task performance^2^ and personality dimensions^21^. Student’s two-tailed t-test with Bonferroni correction confirmed higher PLVs in the Pandemic group for most electrode pairs across all frequency bands (theta: higher PLVs for 802 pairs, lower = 56, alpha: higher = 800, lower = 56, beta-1: higher = 798, lower = 59, beta-2: higher = 794, lower = 90). PLV differences for all four EEG bands are shown in Fig. 3.

#### Transitive reasoning task

Inter-individual patterns of brain activity have been proposed to contribute to differences in cognitive performance in tasks of attention^22^ and working memory^23,24^. Using a transitive reasoning task, which relies on both attention and memory, we next investigated whether connectivity differences between the two groups were associated with differences in cognitive function.

**Figure 4:**
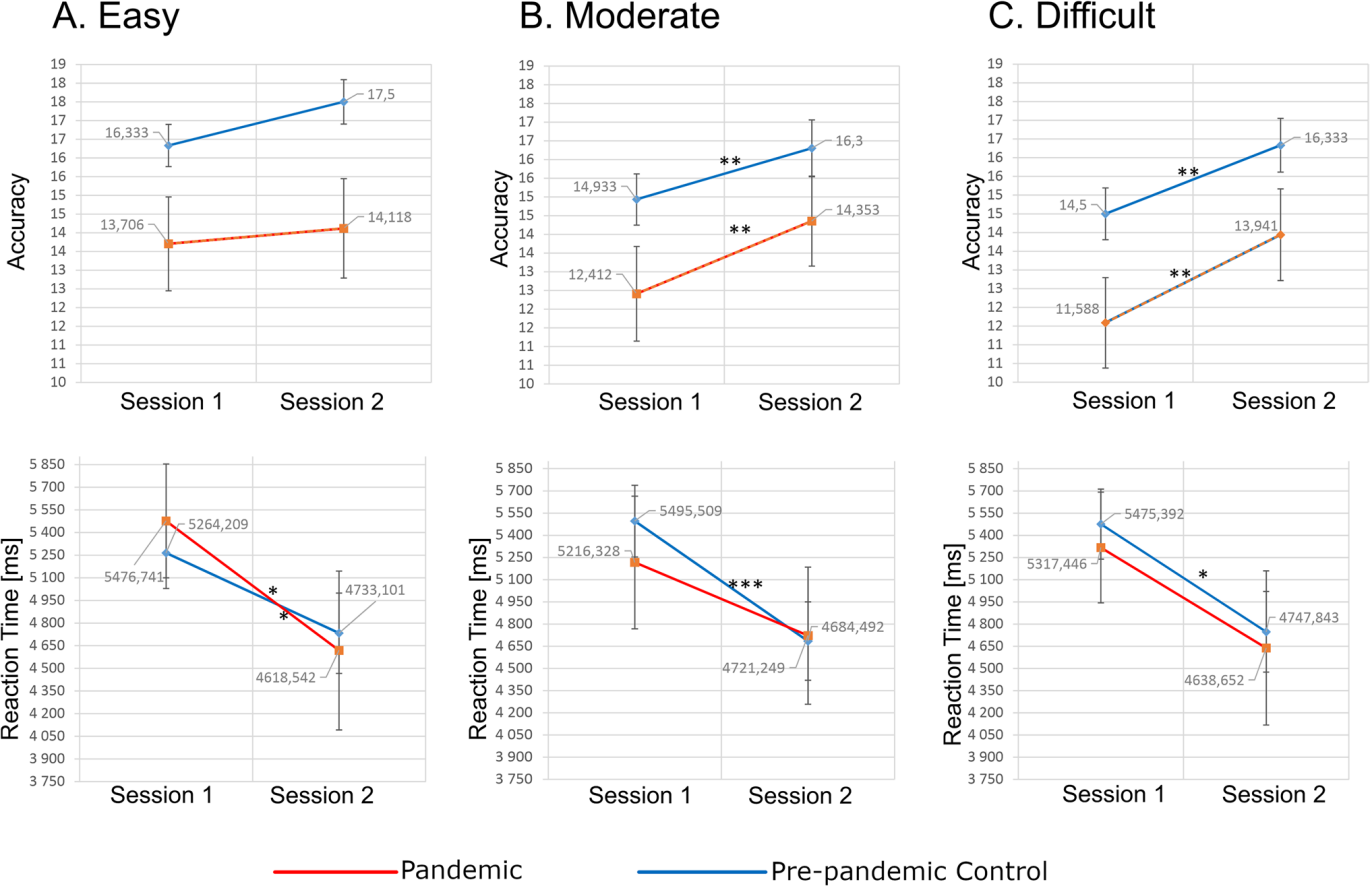
Accuracy and reaction times in a transitive reasoning task. Mean accuracies (upper row) and mean reaction times (lower row) results for Pandemic and Pre-pandemic Control groups in the easy (**A**), moderate (**B**) and difficult (**C**) task variants. The stars above lines joining results of Sessions-1 and Session-2 denote significant differences (*p < 0.05; **p < 0.01; ***p < 0.001) of Chi^2^ post-hoc test followed by Friedman’s test.). Created using JASP 0.14.1 (https://jasp-stats.org).

Participants were tested two times 8–12 weeks apart; Pre-pandemic Control group – before pandemic (Session-1 and 2); Pandemic group – before pandemic (Session-1) and during pandemic (Session-2). Accuracy and reaction times did not differ between the groups for Session-1 or Session-2 (Fig. 4). Friedman ANOVA, and subsequent post hoc analyses, revealed that in both groups accuracy significantly increased in the second vs. first session for moderate and difficult, but not easy, versions of the task. In the Pre-pandemic Control group reaction times were faster, in Session-2, for all versions of the task, whereas in the Pandemic group faster reaction times were found only for the easy task (Fig. 4, Suppl. Inf. Table S3).

Considering that Pre-pandemic Control and Pandemic groups differed on scores of extraversion and EEG connectivity we speculated that this could influence the differences in reaction time between Session-1 and 2.

### Assessment of the impact of COVID-19 lockdown using Pre-pandemic controls matched for personality traits

We attempted to control for personality differences by selecting individuals from the Pre-pandemic Controls to match Pandemics subjects for personality traits, SES, sex and age. We retrospectively analyzed 18 participants and termed this group “Matched Controls” (see Suppl. Inf. Table S4 for details). Matched Controls and Pandemics had similar scores for fear of COVID-19 (median based on all questions for both groups: med = 11.5, Wilcoxon test *p* = 0.75) and similar scores in transitive reasoning task and GCI (see “Effect of pandemic threat on performance in a transitive reasoning task” and “Assessment of impact of pandemic threat on GCI and PLV” sections).

#### Effect of pandemic threat on performance in a transitive reasoning task

**Figure 5:**
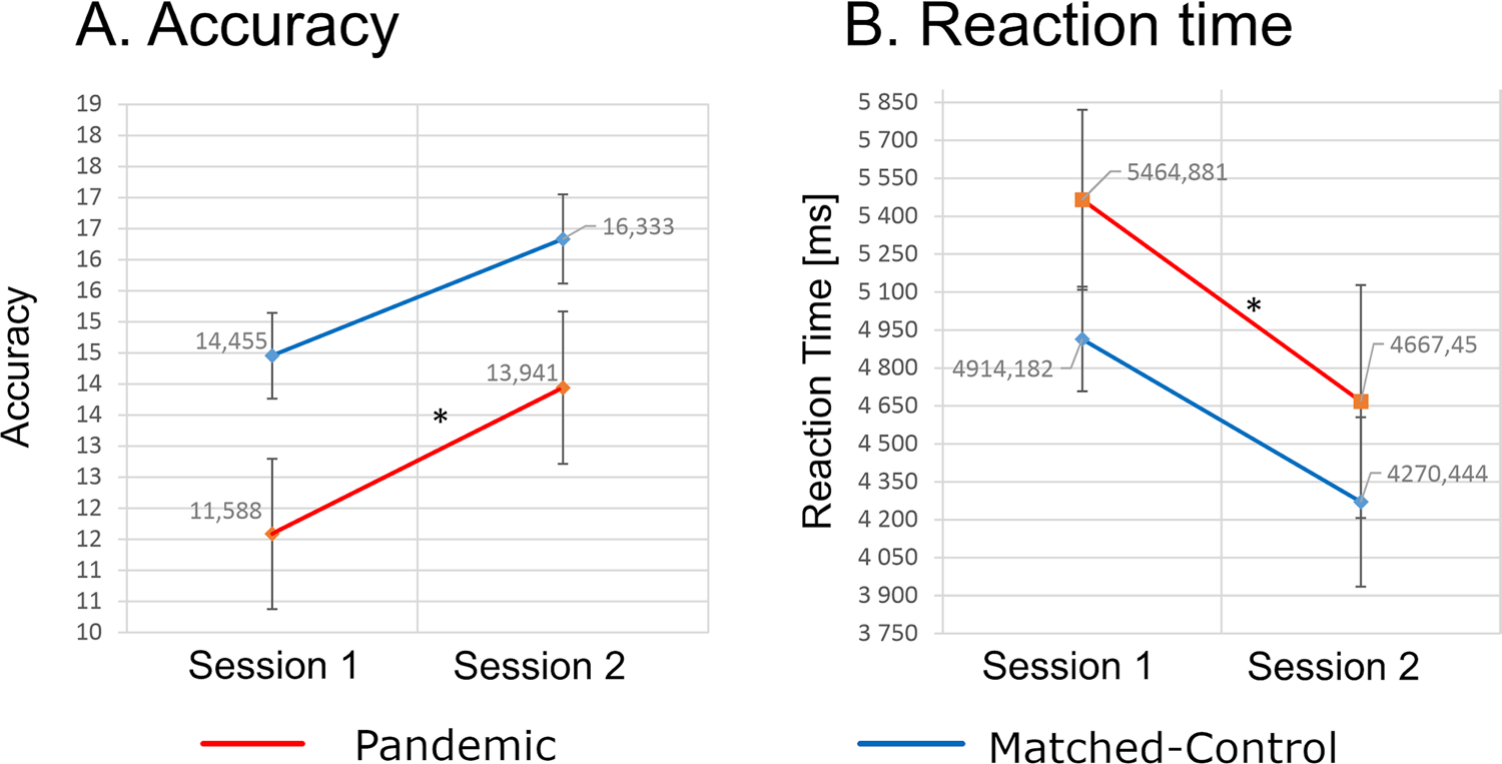
Accuracies and reaction times in transitive reasoning tasks performed in Session-1 and 2. Mean accuracies in difficult task variant (**A**) and mean reaction times in easy task variant (**B**). Stars above lines joining the results of Sessions-1 and Session-2 denote significant differences (*p* < 0.05) of Chi^2^ post-hoc test followed by Friedman’s non-parametric ANOVA. Created using JASP 0.14.1 (https://jasp-stats.org).

Comparison of Pandemics and Matched Controls revealed a significant difference (Friedman test) in accuracy for the difficult task variant, and in reaction times for the easy variant. Post-hoc analyses for sessions and groups did not find significant differences. Within group comparisons (Session-1 vs. Session-2) revealed improved accuracy and shorter reaction times for the Pandemics alone (Fig. 5). Thus, excluding the pandemic threat as a factor underlying these effects in the Pandemics (see Suppl. Inf. Table S5).

To exclude differences in EEG connectivity as a potential factor influencing reaction we compared GCI scores in Pandemics and Matched Controls. We found no group differences for Session-1 for any band (theta: *p* = 0.18; alpha: *p* = 0.18; beta-1: *p* = 0.27; beta-2: *p* = 0.14; one-way ANOVA, post-hoc Tukey–Kramer test) or in the fear of COVID-19 level (*p* = 0.76, Wilcoxon test). Thus, GCI and extraversion were excluded as potential factors accounting for the difference in cognitive performance.

#### Assessment of impact of pandemic threat on GCI and PLV

Repeated measures ANOVA was used to assess the effect of COVID threat on GCI (group*EEG band) for all EEG bands. We found significant differences for group and group*session interactions for all bands (theta: group factor F = 254.49, interaction F = 447.98; alpha: group factor F = 254.06, interaction F = 447.66; beta-1: group factor F = 260.38, interaction F = 457.26; beta-2: group factor F = 241.20, interaction F = 437.63; all significant at *p* < 0.01). Subsequent post-hoc tests showed no difference between the groups in Session-1 (*p* > 0.3), but significant differences in Session-2 for all bands. In Session-2 we observed increased GCI values for all bands in the Pandemics (theta: diff = 0.0178, alpha: diff = 0.0179, beta-1: diff = 0.0182, beta-2: diff = 0.0200; all significant at *p* < 0.01), while for Matched Controls GCI decreased (theta: diff = -0.0126, alpha: diff = -0.0126, beta-1: diff = -0.0121, beta-2: diff = -0.0165; all significant at *p* < 0.01).

**Figure 6:**
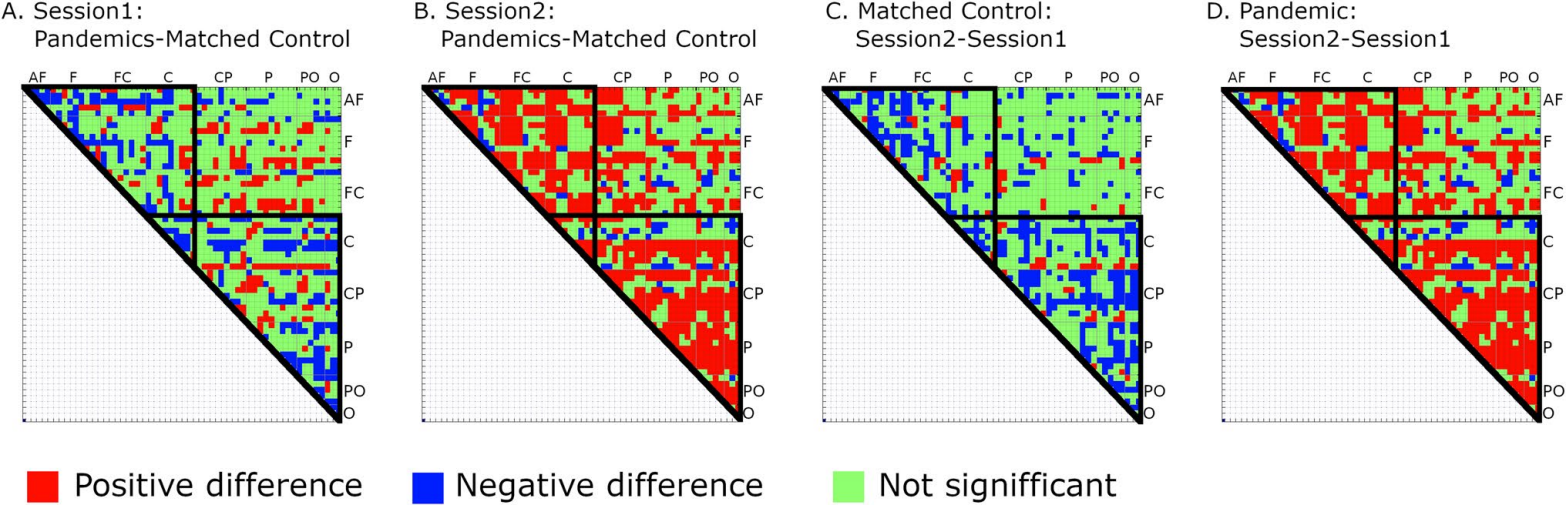
Comparison of differences for all PLV pairs measured between Pandemic and Matched Control groups and between sessions for these groups, in an exemplary beta-2 band. (**A**) Differences between all PLVs for Pandemic and Matched Control groups in Session-1 performed for both groups before the pandemic outbreak; (**B**) the same for Session-2 which was performed for Matched Control group before the pandemic outbreak, and for Pandemic group during lockdown; (**C**) differences between all PLVs measured in Session-2 and Session-1 for Matched Control group, both sessions performed before pandemic outbreak; (**D**) Differences between Session-2 (performed during lock-down) and Session-1 (before the pandemic outbreak) for Pandemic group. Black triangular outlines denote frontocentral and centroparietal PLV values with the most numerous differences. Differences significant at *p* < 0.01, Bonferroni corrected. Created using MATLAB 2020a (The MathWorks, Inc, www.mathworks.com).

The same pattern of connectivity differences was found for all pairs of electrode signals. Figure 6 shows PLV differences (Bonferroni corrected) calculated between the Pandemics and Matched Controls for the beta-2 band for Session-1 and 2 and between both sessions (Fig. SF1 in Suppl. Inf. shows differences for all investigated bands). The strengthening of connectivity measured for signals recorded from most pairs of electrodes was observed in the Pandemic group between Session-1 (before lockdown) and Session-2 (during lockdown) suggests interrelation with the threat of the pandemic.

## Discussion

Here, we attempted to identify if a set of psychological and physiological traits characterized participants who willingly continued training during lockdown. To this end we compared Pandemics with a Pre-pandemic Controls and its subgroup—a Matched Controls paired for personality, sex, and age with the Pandemic group. We found that Pandemics were characterized by higher intensity of extraversion and stronger EEG connectivity but also stable results of cognitive task during lockdown. Notably, these groups did not differ on fear of COVID-19, and thus subjective evaluation of the perceived threat between the groups could not account for the difference we observed. As such, other biopsychological factors should explain the differences between the Pandemics and Control groups.

Stress caused by social isolation^25,26^ and fear can increase brain activation by rerouting mental resources and decreasing efficiency^15^, which in the long-term may lead to anxiety disorders^27,28^. The same stressors are associated with the current pandemic^29^, but the extent of their adverse effects appears to depend on personality traits^30,31^. Psychological investigations report that higher extraversion is associated with lower stress levels^32–34^. This relationship may arise from a reduced physiological response to stress in extraverts^35,36^, their tendency to appraise a situation as less threatening^37^, and positive reinterpretation^38^. Consequently, extraverts might be more optimistic when faced with threats and/or use social engagement as a stress-coping mechanism^39^. Indeed, our results showed that individuals who continued to participate in this study, despite the increased risk of COVID-19, had higher extraversion compared to the controls (comp. Figure 1). Considering that extraversion is associated with a drive for social interaction^29,40,41^ and stimulation^42^, social reward may have been sufficiently motivating in the Pandemic group to outweigh fear of COVID-19.

Other specific traits of the Pandemic group (compared to Pre-pandemic Controls) were higher GCI and stronger PLVs. To date, only a few studies have addressed patterns of EEG connectivity and stress/fear in healthy subjects^16,43^. In particular Alonso and colleagues^43^ showed that stress induced by cognitive task led to increased connectivity in the beta-2 band. This finding is consistent with our observations, but in our case the extent of increased connectivity spanned over all investigated bands probably due to higher and persistent stress caused by the pandemic threat.

Theoretical considerations indicate that strong EEG connectivity in the beta-2 band is highly stable^44,45^, less prone to disturbance^44,46^ and consequently results in less dynamic processing and behavior^47,48^. This lower reconfiguration capability is also in line with our previous investigation which showed that subjects with stronger PLVs exhibited less flexible connection patterns^49^. Negative correlations between the strength of EEG connectivity and performance in attention and memory tasks has been confirmed in ADHD patients^50–52^. These studies showed that strong parietal and occipital connectivity correlated with inattention type ADHD. In the context of the current study, the decision by Pandemics to continue engagement in the study, despite risk, could be reinforced by a lower reconfiguration capability of neuronal networks and subsequently, reduced capacity to adjust their behavior to current environmental conditions^49^. Thus, lower network reconfiguration capacity, combined with higher extraversion appear to be associated with the decision to continue participation in this study. Exposure to a threat and associated stress, could lead to the increased EEG connectivity we observed in the cognitive test session, which took place during lockdown [comp. ^43^]. By contrast, under control conditions (before COVID-19) we observed decreased PLV scores in the second session in both control groups.

Exposure to stress among Pandemics was associated with faster reaction times in a transitive reasoning task compared to Matched Controls (Fig. 5) and no change in reaction time, in its two most difficult variants, compared to Pre-pandemic Controls (Fig. 4). These seemingly contradictory observations may result from strong EEG connectivity specific for Pandemics and Matched Controls. Lower capability of behavioral adjustment caused by strong connectivity^49^ could result in worse performance of Matched Controls under control conditions. Only strong stimulation by pandemic threat allowed network reconfiguration of the Pandemic group leading to outperformance of Matched Controls. Paradoxically, such a mechanism might not necessarily be disadvantageous—in stressful situations strong EEG connectivity and low capability of behavioral adjustment may help to protect cognitive resources against deterioration and subsequent decrease of efficiency, although at the same time it could also result in less adherence to pandemic regulations.

## Methods

### Study design

The study included two groups recruited for an independent, ongoing neurofeedback experiment. Independent analysis yielded that neurofeedback training did not affect neither the cognitive abilities nor the EEG characteristics of the participants who had completed the experiment, and the other group who’s training period happened during lock-down had their two examinations (separated by two months) before awaited training and therefore changes in their behavioral performance were neither expected. None participants from either group reported COVID-19 contagion. Both groups completed psychological assessments and repeated testing of cognitive tasks and resting-state EEG (Session-1 and Session-2, two months apart; Suppl. Inf. Table S6). Participants from the first group who completed all examinations before the pandemic formed the Pre-pandemic Control group, while participants whose' second testing session fell within the lockdown period were qualified to the Pandemic group (in contrast to five subjects who decided to discontinue their engagement after the announcement of a lock-down). The Fear of COVID-19 questionnaire was administered to both groups mean = 55.53 (SD = 12.37) days after the announced lockdown (i.e., between April and May 2020).

In the final step, we aimed at assessment of the impact of the pandemic threat on cognitive and electrophysiological features of brain activity. To this end, we selected from Pre-pandemic Controls participants matching personality traits, sex, age, and socio-economic status (SES) of the Pandemics (the Matched Controls). Next we compared differences between Session-1 and Session-2 for the Pandemics and Matched Controls.

### Participants

The procedures were approved by the Local Bioethics Committee at Nicolaus Copernicus University in Torun. All participants gave their written informed consent to participate in the experiment in accordance with the WMA Declaration of Helsinki – Ethical Principles for Medical Research Involving Human Subjects. All experiments were performed under all relevant guidelines and regulations.

We examined 62 healthy adults (34 women) recruited through announcements at local universities and work agencies. The exclusion criteria were based on neurological screening and questionnaires and included neurological disorders, brain injury, current use of analgesic medication, substance abuse or dependence, and mental disorders. All participants were right-handed and had a normal or corrected-to-normal vision. The mean ± standard deviation of their age was 26.8 ± 4.7 ranging between 23 and 46 years.

The groups and times of their testing are summarized in Supplementary materials in Suppl. Inf. Table S6.

### Personality dimensions

Personality dimensions (i.e., extraversion—E and neuroticism—N) were assessed using the paper-and-pencil individually administered Polish version of the revised Eysenck Personality Questionnaire^5^.

The Polish version^52^ consisted of 106 dichotomous items (yes/no) to assess the three dimensions of personality (extraversion, neuroticism, and psychoticism in E, N, and P scales, respectively) and the tendency to lie or distort responses in a favorable direction (L Scale). We focused on extraversion and neuroticism (E and N Scales). The result of a given scale consisted of the sum of points obtained in response to the questions it comprised, i.e., 23 for E scale (e.g., “Do you enjoy meeting new people?”), and 24 for the N scale (e.g., “Would you call yourself a nervous person”). The Polish version of the EPQ-R was shown to possess good reliability (α = 0.62–0.72, 0.78-0.81, 0.86-0.88, and 0.76–84 for P, E, N, and L scales, assessed in the Polish standardization trials and the four-factor structure. The reliability of the E and N Scales in the current sample was also good (α = 0.71 and 0.74, for E and N scales, respectively).

The raw scores for E and N Scales were converted into standard scores using the following formula (the sten scale): (Z-score × 2) + 5.5, in relation to groups distinguished by sex and age^52^.

### The fear of COVID-19 scale

The computerized, self-administered scale of Fear of COVID-19 (the FCV-19S;^53^; Polish translation: Gola, 2020) was used to assess the fear of coronavirus. The questionnaire consisted of seven items: 1. “I am most afraid of COVID-19”, 2. “It makes me uncomfortable to think about Corona”, 3. “My hands become clammy when I think about Corona”, 4. “I am afraid of losing my life because of Corona”, 5. “When watching news and stories about Corona on social media, I become nervous or anxious”, 6. “I cannot sleep because I’m worried about getting Corona”, 7. “My heart races or palpitates when I think about getting Corona”. Participants responded to each item on a five-point Likert scale (from “strongly disagree” to “strongly agree”). A total score (ranged from 7 to 35) was calculated by summing the scores obtained for all items.

The original version of the questionnaire was shown to present a good internal consistency (α = 0.82) and the one-factor structure (confirmatory factor analyses). Our sample also showed good reliability (Cronbach’s α of 0.839).

### Transitive reasoning task

The laboratory task, which effectively evaluates the effect of threat on cognition, should engage cognitive functions that are vulnerable to anxiety-induced changes (such as working memory and attention). We chose a transitive reasoning test that requires simultaneous processing, maintenance, and manipulation of information, which requires effective attention and working memory capacities^54^.

The version used in the current study was described in detail by Chuderski^55^. Briefly, we used three pairs of Greek letters with greater than or less than symbols. They were displayed for 10 s in the center of the screen, for example: (Ψ > Ω) (Ω > ή) (έ < ή) describing the relationship between the putative values of four different letters. Participants had to deduce the order of the four elements. After 10 s of familiarization, three new pairs appeared on the screen below the three original pairs with only one of them correct. The correct answer should match the guessed arrangement of the original rule. Participants were allowed 10 s for the answer. The task consisted of three conditions: 1. The easiest: where the premises and the elements inside them were arranged linearly from left to right on the basis of "greatest" to "smallest" or vice versa with equal probability; 2. Medium: The same as the easiest but the order of one element was random (keeping the relationship within elements); 3. Difficult: The same as the easiest but the order of two elements was random (keeping the relationship within elements). The whole task consisted of 60 trials.

### EEG recording and preprocessing

Four minutes resting-state eyes open EEG was recorded in both sessions with 128 Ag/AgCl electrodes (Quick Amp; Brain Products GmbH, extended 10–20 system, sampling rate of 1000 Hz), reference at FCz and ground at FPz electrode. The impedance of electrodes was below 10 KΩ. The preprocessing included 0.5–70 Hz filtering, baseline correction, exclusion of 1 s data segments containing artifacts, and independent component analysis (ICA). The identified eye movement and muscle artifacts components were removed (maximum 10% of all ICA components).

### Connectivity analyses

Spontaneous brain activity of the large-scale EEG and fMRI networks can predict cognitive performance^49,56,57^ we seized the opportunity to investigate the effect of the threat on the EEG connectivity. As a proxy we used phase-locking value [PLV, ^58^]. PLV is used for connectivity estimation in EEG/MEG studies^59–62^. PLV does not depend on spectral power and is robust to noisy signals^63^. Zero-phase correlations present the PLV provide information enabling prediction of the task results, not possible otherwise^64^. Methods preserving zero-phase correlations (PLV and AEC) significantly relate to the results of fMRI analyses unlike others^65^.

To compute PLV in a given frequency range, we filtered the EEG data using a two-sided finite impulse response filter and then subjected them to a Hilbert transform for computation of the instantaneous amplitude and phase. Only the phase component was used for PLV computation. The PLVs were calculated for 1-s non-overlapping epochs. The number of epochs was limited to the shortest EEG resting-state signal which remained after preprocessing, i.e. to 140 epochs. For the purpose of analyses, epochs were group averaged yielding the same number of samples (140) independently of the group size. As a general measure of connectivity in the given frequency band, we averaged PLVs of all electrode pairs (Global Connectivity Index—GCI). Similar global measures of EEG connectivity were proposed as potential biomarkers of different abnormal brain states^66,67^.

Due to high correlations of the signals collected from closely located electrodes in a 128 cap set we used subset matching standard positions of the 64 electrode cap (AF7, AF3, AFz, AF4, AF8, F7, F5, F3, F1, Fz, F2, F4, F6, F8, FT7, FC5, FC3, FC1, FCz, FC2, FC4, FC6, FT8, T7, C5, C3, C1, Cz, C2, C4, C6, T8, TP7, CP5, BP3, CP1, CPz, CP2, CP4, CP6, TP8, P7, P5, P3, P1, Pz, P2, P4, P6, P8, PO7, PO3, POz, PO4, PO8, O1, Oz and O2) except for the 7 electrodes located near jaws and neck, usually most contaminated by muscle artifacts.

### Assessment of the impact of pandemic threat on the cognitive and electrophysiological processes

The assessment was performed by comparing within-group differences between Session 2 and Session 1 in the Pandemic and Matched Control groups. The Matched Control group consisted of participants selected from the Pre-pandemic Control group that matched the Pandemic subjects in terms of personality dimensions, sex, age, and socioeconomic status.

### Statistical methods

Data were tested for normality by the Kolmogorov–Smirnov test and checked for the presence of outliers. The values deviating from the mean for more than three standard deviations were removed. Comparison of the groups were conducted using two-tailed two-sample t-tests or ANOVA. In cases of small samples, the Man-Whitney test and Friedman ANOVA with Chi square post-hoc test, were used. All PLV analyses were performed on single trial data. Results were corrected for false positives using Bonferroni adjustment where appropriate unless otherwise stated. Analyses and figures were created using MATLAB 2020a (connectivity analyses), R 4.0.4 (personality dimensions and SES analyses) and JASP (behavioral statistics).

## Supplementary Information

Supplementary Information.
