# Supplementary material for: Stronger connectivity and higher extraversion protect against stress-related deterioration of cognitive functions
Source: Sci Rep. 2021 Aug 31;11:17452. doi: 10.1038/s41598-021-96718-5 (PMC8408208; doi:10.1038/s41598-021-96718-5)
Supplement: Supplementary file 1 — Supplementary Information. [file 41598_2021_96718_MOESM1_ESM.docx]

**Supplementary Information**

**Table S1.** Descriptive statistics for the fear of COVID-19 and personality dimensions (Extraversion and Neuroticism) for Pre-pandemic Control and Pandemic groups.

| Group/Statistics | Scores | Min-Max | M(SD) | Skew | Kurtosis |
| --- | --- | --- | --- | --- | --- |
| *Pre-pandemic Control, n = 44* | | | | | |
| Fear of COVID-19 | Raw | 7-24 | 13.39(4.51) | 0.621 | 0.066 |
| Extraversion | Raw | 2-22 | 11.73(5.5) | 0.155 | -0.964 |
|  | Sten | 1-10 | 4.05(2.51) | 0.615 | -0.695 |
| Neuroticism | Raw | 1-22 | 9.91(5.96) | 0.435 | -1.03 |
|  | Sten | 1-9 | 4.84(2.13) | 0.082 | -0.706 |
| *Pandemic, N = 18* | | | | | |
| Fear of COVID-19 | Raw | 7-27 | 13.14(5.19) | 1.53 | 2.933 |
| Extraversion | Raw | 4-17 | 15.22(4.88) | -0.988 | 0.099 |
|  | Sten | 1-9 | 5.44(2.55) | -0.351 | -0.659 |
| Neuroticism | Raw | 0-22 | 11.44(6.87) | -0.217 | -1.286 |
|  | Sten | 1-9 | 5.06(2.46) | -0.312 | -0.466 |

**Table S2.** Descriptive statistics for SES differentiated by groups.

| Socio-economic status (**SES**) | | | | | |
| --- | --- | --- | --- | --- | --- |
| **Pre-pandemic Control**  n=44, 22 females, age: 26.41, SD = 4.67 | *place of residence* | more than 200,000 residents | between 50,000 and 200,000 residents | 5,000 or fewer residents | no information |
|  |  |  |  |  |  |
|  | *%* | 84.09 | 4.55 | 9.09 | 2.27 |
|  | *education level* | a university degree (bachelor's or master's degree) | the Polish equivalent of a high school degree | primary education | no information |
|  | *%* | 52.27 | 45.45 | - | 2.27 |
|  | *occupational status* | university student | full and part time | business owner | unemployed |
|  | *%* | 54.54 | 38.64 | 4.55 | 2.27 |

| SES | | | | | |
| --- | --- | --- | --- | --- | --- |
| **Pandemic**  n=18, 12 females, age: M = 26.06, SD = 4.32 | *place of residence* | more than 200,000 residents | between 50,000 and 200,000 residents | 5,000 or fewer residents | no information |
|  |  |  |  |  |  |
|  | *%* | 66.66 | 5.55 | 5.55 | 22.2 |
|  | *education level* | a university degree (bachelor's or master's degree) | the Polish equivalent of a high school degree | primary education | no information |
|  | *%* | 50 | 50 | - | - |
|  | *occupational status* | university student | full and part time | business owner | unemployed |
|  | *%* | 61.11 | 38.89 | - | - |

**Table S3**. Comparison of Transitive Reasoning task results between Pre-pandemic Control and Pandemic groups. Non-parametric ANOVA analyses showed significant results for reaction times and accuracy.

| Post Hoc Comparisons Condition Easy | | Reaction Time | | | |
| --- | --- | --- | --- | --- | --- |
|  |  | Mean Difference | SE | t | p _holm_ |
| Session 1,Pandemic | Session 1,Pre-pandemic Control | 71.464 | 508.336 | 0.141 | 1.000 |
|  | Session 2,Pandemic | 844.464 | 260.061 | 3.247 | 0.013 |
| Session 1,Pre-pandemic Control | Session 2,Pre-pandemic Control | 513.365 | 189.921 | 2.703 | 0.049 |
| Session 2,Pandemic | Session 2,Pre-pandemic Control | -259.635 | 508.336 | -0.511 | 1.000 |

*Note.*  Bonferroni adjusted confidence intervals.

| Post Hoc Comparisons Condition Moderate | | Accuracy | | | | Reaction Time | | | |
| --- | --- | --- | --- | --- | --- | --- | --- | --- | --- |
|  |  | Mean Difference | SE | t | p _holm_ | Mean Difference | SE | t | p _holm_ |
| Session 1,Pandemic | Session 1,Pre-pandemic Control | -2.371 | 1.369 | -1.732 | 0.266 | -561.863 | 489.047 | -1.149 | 0.767 |
|  | Session 2,Pandemic | -3.538 | 1.369 | -2.585 | 0.074 | 439.827 | 264.524 | 1.663 | 0.414 |
| Session 1,Pre-pandemic Control | Session 2,Pre-pandemic Control | -1.167 | 0.590 | -1.978 | 0.217 | 786.281 | 193.181 | 4.070 | 0.001 |
| Session 2,Pandemic | Session 2,Pre-pandemic Control | -1.788 | 1.369 | -1.306 | 0.393 | -215.409 | 489.047 | -0.440 | 1.000 |

*Note.*  Bonferroni adjusted confidence intervals.

| Post Hoc Comparisons Condition Difficult | | Accuracy | | | | Reaction Time | | | |
| --- | --- | --- | --- | --- | --- | --- | --- | --- | --- |
|  |  | Mean Difference | SE | t | p _holm_ | Mean Difference | SE | t | p _holm_ |
| Session 1,Pandemic | Session 1,Pre-pandemic Control | -2.971 | 1.326 | -2.240 | 0.087 | -334.847 | 503.136 | -0.666 | 1.000 |
|  | Session 2,Pandemic | -2.125 | 0.713 | -2.982 | 0.019 | 605.175 | 332.081 | 1.822 | 0.333 |
| Session 1,Pre-pandemic Control | Session 2,Pre-pandemic Control | -1.733 | 0.521 | -3.330 | 0.009 | 731.836 | 242.518 | 3.018 | 0.025 |
| Session 2,Pandemic | Session 2,Pre-pandemic Control | -2.579 | 1.326 | -1.945 | 0.114 | -208.186 | 503.136 | -0.414 | 1.000 |

*Note.*  Bonferroni adjusted confidence intervals.

**Table S4**. Descriptive statistics for Fear of COVID-19, personality dimensions (Extraversion and Neuroticism), and SES for the Matched Control group.

| **Matched Control**, N = 18, females = 12, age: M = 26, SD = 3.90 | | | | | |
| --- | --- | --- | --- | --- | --- |
| Measures/Statistics | scores | Min-Max | M(SD) | Skew | Kurtosis |
| Fear of COVID-19 | raw | 7-18 | 12.17(3.35) | .377 | -1.126 |
| Extraversion | raw | 6-20 | 14.06(4.44) | -.459 | -.989 |
|  | sten | 1-8 | 4.83(2.04) | -.029 | -.762 |
| Neuroticism | raw | 1-20 | 10.33(5.48) | .210 | -.900 |
|  | sten | 1-8 | 4.83(1.89) | -.263 | -.455 |
|  | | | | | |
| SES | *place of residence* | more than 200,000 residents | between 50,000 and 200,000 residents | 5,000 or fewer residents | no information |
|  | *%* | 77.78 | 5.56 | 11.11 | 5.56 |
|  | *education level* | a university degree (bachelor's or master's degree) | the Polish equivalent of a high school degree | primary education | no information |
|  | *%* | 66.67 | 33.33 | - | - |
|  | *occupational status* | university students | full and part time | own business | unemployed |
|  | *%* | 55.56 | 38.89 | - | 5.56 |

**Table S5** Comparison of Transitive Reasoning task results between Matched Control and Pandemic groups. Non-parametric ANOVA analyses showed significant results for reaction times and accuracy.

| Friedman ANOVA | Accuracy | | Reaction Time | |
| --- | --- | --- | --- | --- |
|  | χ2 | p | χ2 | p |
| Easy | 0.053 | 0.819 | 10.704 | **0.001** |
| Moderate | 1.190 | 0.275 | 1.815 | 0.178 |
| Difficult | 8.167 | **0.004** | 3.000 | 0.083 |

| Post Hoc Comparisons Condition Easy | | Reaction Time | | | |
| --- | --- | --- | --- | --- | --- |
|  |  | Mean Difference | SE | t | p _holm_ |
| Session 1,Matched Control | Session 1,Pre-pandemic Control | -484.571 | 694.258 | -0.698 | 1.000 |
|  | Session 2,Matched Control | 692.979 | 346.316 | 2.001 | 0.282 |
| Session 1,Pandemic | Session 2, Pandemic | 844.464 | 287.150 | 2.941 | **0.042** |
| Session 2, Matched Control | Session 2, Pandemic | -333.085 | 694.258 | -0.480 | 1.000 |

*Note.*  Bonferroni adjusted confidence intervals.

| Post Hoc Comparisons Condition Easy | | Accuracy | | | |
| --- | --- | --- | --- | --- | --- |
|  |  | Mean Difference | SE | t | p _holm_ |
| Session 1,Matched Control | Session 1,Pre-pandemic Control | 2.892 | 1.835 | 1.576 | 0.377 |
|  | Session 2,Matched Control | -1.455 | 0.806 | -1.806 | 0.332 |
| Session 1,Pandemic | Session 2, Pandemic | -2.125 | 0.668 | -3.181 | **0.023** |
| Session 2, Matched Control | Session 2, Pandemic | 2.222 | 1.835 | 1.211 | 0.471 |

*Note.*  Bonferroni adjusted confidence intervals.

**Table S6.** Groups participating in the study. Cross denotes type and date of examination

| **Group** | Socio-economic status (**SES**) | **Test**  (session including EEG, resting-state, cognitive task) / Questionare (Fear of COVID-19 ) | Sep -Oct 2019 | Apr-May 2020  (lock -down) |
| --- | --- | --- | --- | --- |
| **Pre-pandemic Control**  n=44, 22 females, age: 26.41, SD = 4.67 | *a) place of residence:* 84.09% - cities with more than 200,000 residents; 4.55% - cities between 50,000 and 200,000; 9.09% - locales/cities with 5,000 or fewer; 2.27% - no information  *b) education level:* 52.27% - a university degree (bachelor's or master's degree); 45.45% - the Polish equivalent of a high school degree; 2.27% - no information  *c) occupational status:* 54.54% - university students; 38.64% - full time or part time; 4.55% - own business; 2.27%- no information | **Session 1** | X |  |
|  |  | **Session 2** | X |  |
|  |  | Fear of COVID-19 **questionnaire** |  | X |
| **Pandemic**  n=18, 12 females, age: M = 26.06, SD = 4.32 | *a) place of residence:* 66.66% - cities with more than 200,000 residents; 5.55 % - cities between 50,000 and 200,000; 5.55 % - locales/cities with 5,000 or fewer; 22.22% - no information  *b) education level:* 50% - a university degree (bachelor's or master's degree); 50% - the Polish equivalent of a high school degree  *c) occupational status:* 61.11% - university students; 38.89% - full time | **Session 1** | X |  |
|  |  | **Session 2** |  | X |
|  |  | Fear of COVID-19 **questionnaire** |  | X |

**Figure SF1**


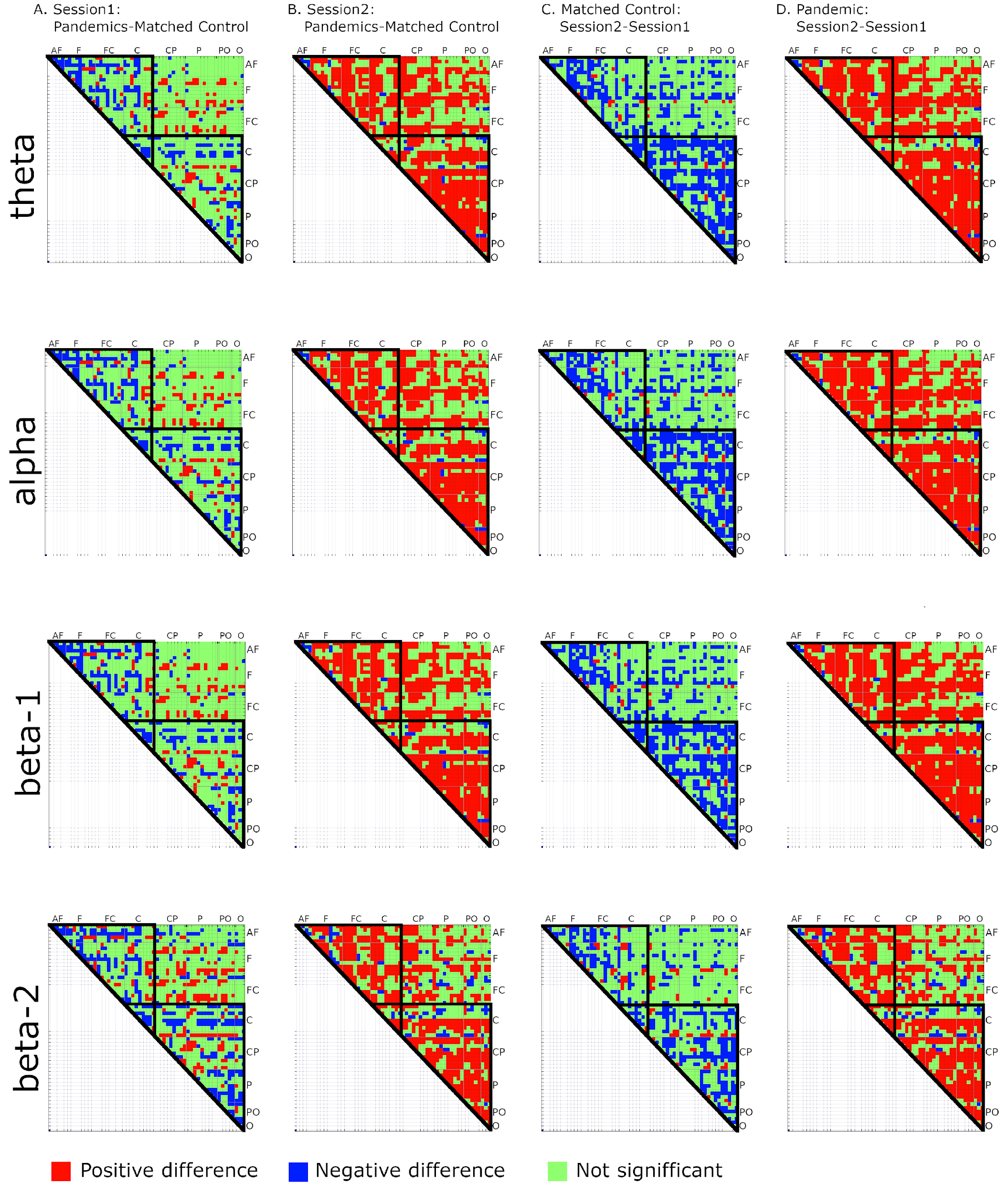


**Fig. SF1**. Differences measured for all PLV pairs (described on the axes of diagrams) between Pandemic and Matched Control groups and between session 1 and 2 for these groups, in all investigated bands. **A.** Positive (red) and negative (blue) differences between PLVs measured for all signal pairs in Pandemic and Matched Control groups in Session-1 performed for both groups before the pandemic outbreak; **B**. The same for Session-2 which was performed for Matched Control group before the pandemic outbreak, and for Pandemic group during lockdown; **C.** Differences between all PLVs measured in Session-2 and Session-1 for Matched Control group, both sessions performed before pandemic outbreak; D. Differences between Session-2 (performed during lock-down) and Session-1 (before the pandemic outbreak) for Pandemic group. Black triangular outlines denote largest PLV values measured within frontocentral and centroparietal connections. Differences significant at p<0.01, Bonferroni corrected. Each pixel denotes difference between PLV values measured for connection within AF (anterofrontal), F (frontal), FC (frontocentral), C (central), CP (centroparietal), P (parietal), PO (posterooccipital), O (occipital) pairs of signals. Created using MATLAB 2020a (The MathWorks, Inc, www.mathworks.com).
